# Air Pollutants in Metropolises of Eastern Coastal China

**DOI:** 10.3390/ijerph192215332

**Published:** 2022-11-20

**Authors:** Mao Mao, Liuxintian Rao, Huan Jiang, Siqi He, Xiaolin Zhang

**Affiliations:** 1 School of Atmosphere and Remote Sensing, Wuxi University, Wuxi 214105, China; 2 Key Laboratory for Aerosol-Cloud-Precipitation of China Meteorological Administration, School of Atmospheric Physics, Nanjing University of Information Science & Technology, Nanjing 210044, China

**Keywords:** particulate matter, air pollutants, eastern coastal China, metropolises

## Abstract

Recently released hourly particular matter (PM:PM_2.5_ and PM_10_) and gaseous pollutants (SO_2_, NO_2_, CO, and O_3_) data observed in Qingdao, Hangzhou, and Xiamen from 2015 to 2019 were utilized to reveal the current situation of air pollution over eastern coastal China. The PM pollution situation over the three metropolises ameliorated during studied period with the concentrations decreasing about 20–30%. Gas pollutants, excepting SO_2_, generally exhibit no evident reduction tendencies, and a more rigorous control standard on gaseous pollutants is neededEven for the year 2018 with low pollution levels among the study period, these levels (<10% of PM_2.5_, <6% of PM_10_, and <15% of O_3_) surpass the Grade II of the Chinese Ambient Air Quality Standard (CAAQS) over these metropolises of eastern coast China. No matter in which year, both SO_2_ and CO concentrations are always below the Grade-II standards. According to the comparative analysis of PM_2.5_/PM_10_ and PM_2.5_/CO during episode days and non-episode days, the formation of secondary aerosols associated with stagnant weather systems play an important role in the pollutant accumulation as haze episodes occurred. The stronger seasonal variations and higher magnitude occur in Qingdao and Hangzhou, while weaker seasonal variations and lower magnitudes occur in Xiamen. In Qingdao and Hangzhou, PM, NO_2_, SO_2_, and CO show relatively high levels in the cold wintertime and low levels in summer, whereas O_3_ shows a completely opposite pattern. Xiamen exhibits high levels of all air pollutants except O_3_ in spring due to its subtropical marine monsoon climate with mild winters. According to the back trajectory hierarchical clustering and concentration weighted trajectory (CWT) analysis, the regional transmission from adjacent cities has a significant impact on the atmospheric pollutant concentrations under the control of the prejudiced winds. Thus, besides local emission reduction, strengthening regional environmental cooperation and implementing joint prevention are effective measures to mitigate air pollution in the eastern coastal areas of China.

## 1. Introduction

Air pollution poses serious threats to human health and sustainable development of the economy and society [1,2,3]. With increasing urbanization and rapidly expanding economic development, China has been experiencing severe air pollution in recent decades, which is one of the foremost environmental concerns and social problems in China [4,5,6,7,8,9]. High concentrations of atmospheric particulate matter (PM) and/or gaseous pollutant will raise the risk of cardiovascular and respiratory system illness in the elderly and reduce neonatal survival [10,11,12]. Anthropogenic activities, including coal combustion, vehicle emissions, roadwork and construction-site dust emissions are leading factors for the temporal–spatial variation of air pollutant [13,14,15]. Many relevant reports and studies of air quality chiefly focused on the megacities and some hot spot city clusters of eastern and central China, such as the North China Plain (NCP), the Yangtze River Delta (YRD), and the Pearl River Delta (PRD), especially in the winter [16,17,18,19,20].

To tackle and control air pollution, the State and local government departments have formulated and promulgated a series of laws, regulations and standards. Starting from January 2013, the Chinese Ministry of Environmental Protection has granted access to the official revisions of the concentrations of six criteria air pollutants, including PM_2.5_ (PM with an aerodynamic diameter less than 2.5 μm), PM_10_ (PM with an aerodynamic diameter less than 10 μm), sulfur dioxide (SO_2_), nitrogen dioxide (NO_2_), carbon monoxide (CO), and ozone (O_3_), which are monitored in some major cities. The continuous monitoring data of air pollutants facilitate us to explore their daily, seasonal and inter-annual variabilities.

**Figure 1:**
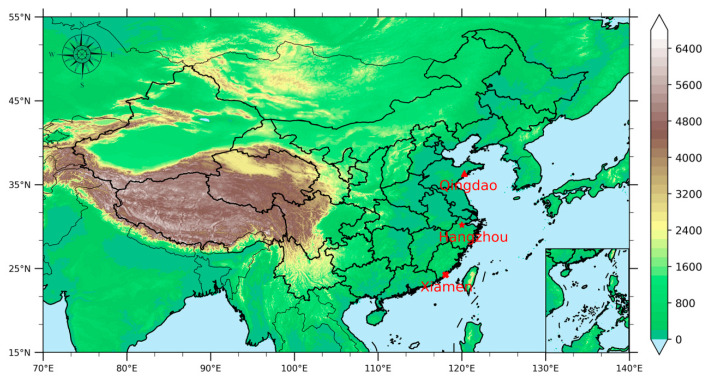
Locations of three metropolises along Eastern costal China. The altitude values are displayed in color.

We focus on the three second-tier coastal cities in east China, including Qingdao, Hangzhou and Xiamen, which are second only to Shanghai, the first-tier city. Three red solid markers denote the locations in Figure 1. Qingdao (35.35–37.09° N, 119.30–121.00° E) is situated on the eastern coast of NCP region and is the most developed city in the Shandong Peninsula. It is categorized as being in a warm temperate zone monsoon climate. According to the 2020 Statistical Bulletin on National Economic and Social Development [21], Qingdao covers an area of around 11,293 km^2^ with a population of 10.1 million and an occurrence of 3.1 million vehicles. Its economy is dominated by light industries and services, and the coal is the primary energy source with an annual consumption of 13.66 million tons. Qingdao is undergoing rapid urbanization and economic development: on one hand, the urbanization rate reaches 69.99%; on the other hand, the gross domestic product (GDP) exceeds 1240 billion CNY, ranking first in Shandong Province and 13th in China in 2020. Hangzhou (29.11–30.33° N, 118.21–120.30° E), lying along the south of the YRD and the Qiantang River Basin with complex and diverse terrain, is the capital city of Zhejiang province. It owns an area of 16,850 km^2^ and a population of 11.94 million [21]. Hangzhou’s GDP ranks among the top eight in China for the first time by the end of 2020. Traditional heavy industries are being replaced by other less polluting manufacturing step by step, such as high-technology industry, the strategic emerging industry, and the equipment manufacturing industry. Internet economy has become a new economic growth point since the beginning of the 21st century. Xiamen Bay (24.23–24.54° N, 117.53–118.26° E), located in the southeast of Fujian Province, is a special economic zone in China approved by the State Council. With 5.16 million inhabitants and 1.7 million vehicles, Xiamen achieves a regional GDP of 638 billion CNY in 2020 [21]. Belonging to subtropical marine monsoon climate, it is a regional hub port along the southeast coast characterized by export processing industries and ocean freight forwarding service. Ship emissions from heavy oil combustion have a certain impact on local air quality. Owning to urbanization with high population density, unprecedented levels of economic growth, and high emission densities of air pollutants such as PM_2.5_, black carbon, and polycyclic aromatic hydrocarbons, the above three second-tier cities have become hot spots in the eastern coastal areas of China.

At present, only a few studies on the impact of anthropogenic and natural factors on PM or trace gases are sporadically conducted in a single city or seasons with higher levels of air pollution [22,23,24], whereas joint studies on the long-term trends of the temporal-spatial pattern of the PM and trace gases for the three eastern coastal metropolises are still scarce so far. In this study, we first comprehensively displayed the characterization and source distribution of air pollution by comparative analysis of Qingdao, Hangzhou and Xiamen during 2015–2019. The main objective of the present study is to understand spatio-temporal distribution and possible causes of six pollutants by discussing the annual, seasonal, and daily variation characteristics, the relationships among the pollutants, and concentrations dependent on local emissions and regional transportation. The relevant knowledge obtained in this study serves as the scientific basis for assessing the air pollution situation and provides references for formulating future urban air pollution control measures on East China.

We give detailed descriptions about the observational data and methodology used in Section 2, and present the main results, focusing on comparisons of temporal variation in Section 3. We will also attempt to understand the pollutant source of the target cities based on a backward trajectory and CWT analysis. A summary is given in Section 4.

## 2. Data and Methodology

### 2.1. Data Source

All data for monitoring national air quality, including particulate matter and gaseous pollutants, are provided by the China National Environmental Monitoring Centre [25]. Recent five-year data from 1 January 2015 to 31 December 2019 are utilized for our analysis. Automatic monitoring systems are installed in the National Environmental Monitoring sites of each city across China, measuring ambient mass concentrations of PM_2.5_, PM_10_, SO_2_, CO, NO_2,_ and O_3_ based on China Environmental Protection Standards (HJ655-2013). Three metropolises, i.e., Qingdao, Hangzhou and Xiamen, are considered in our study, being significant second-tier cities after Shanghai in eastern costal China (Figure 1). The hourly concentrations of air pollutants for each city are averaged from enough monitoring sites within this city, representing mean levels of air qualities, whereas daily, seasonal and annual average concentrations are calculated by averaging the hourly data from all of the monitoring stations unless more than 16 h valid data are released.

Designed as a mix of urban sites (the majority) and background sites (a few) for the point layout of ambient air quality monitoring, presently there are 9, 11, and 3 national automated monitoring sites in Qingdao, Hangzhou and Xiamen, respectively (Table S1). The environmental conditions near the sampling sites, including underlying surface and characteristics emission sources are relatively stable. The mass concentrations of PM_2.5_ and PM_10_ were measured using the Tapered Element Oscillating Micro-balance analyzers. Levels of SO_2_, NO_2_, O_3_, and CO were measured using the ultraviolet fluorescence method, the chemiluminescence method, the ultraviolet spectrophotometry method, and the non-dispersion infrared absorption method (or the gas filter correlation infrared absorption method), respectively. The quality assurance and controls of the state-controlled monitoring stations were reported in previous studies [26,27].

### 2.2. Methods

#### 2.2.1. Trajectory Clustering

The TrajStat model was developed in the Center for Atmosphere Watch and Services (CAWAS) and Chinese Academy of Meteorological Sciences (CAMS). Corresponding meteorological data with a 1° × 1° grid used in the TrajStat model was acquired from the Global Data Assimilation System (GDAS) provided by National Centers for Environmental Prediction (NCEP), which can be downloaded from the Hybrid Single-Particle Lagrangian Integrated Trajectory (HYSPLIT) website [28]. The 72-h backward trajectories were run four times per day at starting times of 0:00, 06:00, 12:00, and 18:00 UTC (8:00, 14:00, 20:00, and 02:00 LT, respectively) with a starting height of 500 m above ground level (agl). The Grade II of Chinese Ambient Air Quality Standard (CAAQS) for daily mean of PM_2.5_ is 75 µg/m^3^, thus, daily average PM_2.5_ > 75 µg/m^3^ is defined as PM_2.5_ episode days. Cluster analyses were carried out on the basis of the hierarchical clustering method on episode days and non-episode days, respectively [29].

#### 2.2.2. Concentration Weight Trajectory (CWT) Model

CWT analysis method can quantitatively give the average weight concentration of each grid by calculating the weight concentration of the trajectory. The values of daily mean PM_2.5_ concentrations for three cities in 2018 are adopted in the CWT maps. The CWT model is applied to weight-back trajectories (four times per day, at 0:00, 6:00, 12:00, and 18:00 UTC) with related PM_2.5_ concentrations for unraveling the relative contribution of different sources-areas [30,31,32,33]. Areas with high CWT values are considered as potential geographic origins of PM_2.5_. The zone of concern is divided into small equal grid cells (*i* × *j*). The geographical field is divided into grid cells representing an area of 0.5° × 0.5°. The CWT is defined as follows:

(1) $$C_{ij}=\frac{\sum_{h=1}^{M}C_{h}\times \tau_{ijh}}{\sum_{h=1}^{M}\tau_{ijh}}\times W(n_{ij})$$

where *i* and *j* denote the latitude and longitude, *C_ij_* is designed as the average weight concentration of the trajectory *h* in the *ij*th cell, *h* represents the index of the trajectory, *M* represents the total number of trajectories, *C_h_* denotes PM_2.5_ concentrations in the trajectory *h* through *ij*th cell, and τ*_ijh_* is the time that trajectory *h* resides in the *ij*th cell. The *n_ij_* is on behalf of the total number of endpoints that fall in the *ij*th cell. The arbitrary weighting function *W*(*n_ij_*) is applied to the CWT method for reducing the uncertainty in cells when *n_ij_* is lower than three times of average number of trajectory endpoints (*n_ave_*) in each cell [34,35]. The *W*(*n_ij_*) is described as follows:

(2) $$W(n_{ij})=\left\{ \begin{array}{l} 1.00,3n_{ave}<n_{ij}\\ 0.70,1.5n_{ave}<n_{ij}\leq 3n_{ave}\\ 0.40,n_{ave}<n_{ij}\leq 1.5n_{ave}\\ 0.20,n_{ij}\leq n_{ave} \end{array}\right.$$

The study domain is in the range of 25–65° N and 90–140° E for Qingdao (20–60° N for Hangzhou and 15–55° N for Xiamen), which contains more than 95% of areas covered by all the paths. The total number of endpoints in each city is about 105120, and the area covered by the trajectories is divided into 8000 grid cells (0.5° × 0.5°). Hence, each cell owns an average of 13 trajectory endpoints, that is, the *n*_ave_ is equal to 13. The CWT analysis is run by the MeteoInfo software-TrajStat Plugin [30].

## 3. Results and Discussion

### 3.1. Situation and Implication of Air Pollutants

**Figure 2:**
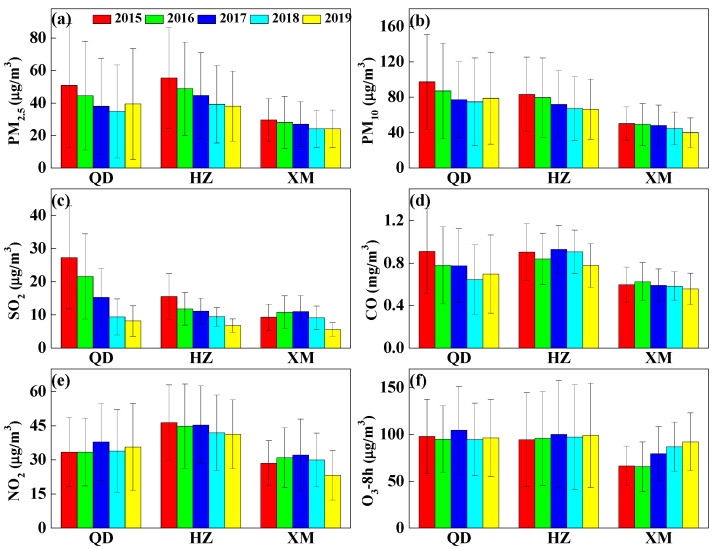
Inter-annual variations of PM_2.5_ (**a**), PM_10_ (**b**), SO_2_ (**c**), CO (**d**), NO_2_ (**e**), and 8 h peak O_3_ (**f**). in Qingdao (QD), Hangzhou (HZ), and Xiamen (XM) during 2015–2019. The error bar represents standard deviation.

The citywide average concentration is obtained by averaging the concentrations of all sites in every city. Figure 2 depicts inter-annual variations of mean mass concentrations of six air pollutants in Qingdao, Hangzhou and Xiamen from 2015 to 2019. The statistics of PM_2.5_, PM_10_, SO_2_, CO, NO_2_, and 8 h peak O_3_ (8 h-averaged O_3_) for the three metropolises of eastern coast China are listed in Table S2. The yearly mean mass concentrations of PM_2.5_ decrease gradually from 55 ± 31 and 30 ± 13 μg/m^3^ in 2015 to 38 ± 21 and 24 ± 11 μg/m^3^ in 2019 over Hangzhou and Xiamen, respectively. Nevertheless, in Qingdao, PM_2.5_ mean mass concentration is reduced from 2015 (51 ± 38 μg/m^3^) to 2018 (35 ± 29 μg/m^3^), and shows an increase in 2019 (40 ± 34 μg/m^3^). The inter-annual variation trends of PM_10_ are similar to PM_2.5_, and their yearly average mass concentrations vary in ranges of 75–97, 65–83, and 40–50 μg/m^3^ in Qingdao, Hangzhou and Xiamen, respectively. Obviously, the standard deviation of PM_2.5_ and PM_10_ in Qingdao is almost two or three times higher than in Xiamen as shown in Table S2, exhibiting large variances of PM in Qingdao and small variances of PM in Xiamen. As compared to the Grade II of Chinese Ambient Air Quality Standards (CAAQS) for the annual mean (35 μg/m^3^ for PM_2.5_ and 70 μg/m^3^ for PM_10_), some PM_2.5_ and PM_10_ levels have exceeded the limit for Qingdao and Hangzhou, indicating a high health risk. The mean SO_2_ mass concentrations lessen from 27 ± 15 and 16 ± 7 μg/m^3^ in 2015 to 8 ± 5 and 7 ± 2 μg/m^3^ in 2019 for Qingdao and Hangzhou, respectively, whereas it is between 5 ± 2 and 11 ± 5 μg/m^3^ in Xiamen during 2015–2019. In Qingdao, Hangzhou and Xiamen, annual mean mass concentrations of NO_2_ and O_3_ are observed in ranges of 33–38 and 95–104, 41–46 and 94–100, and 23–32 and 66–92 μg/m^3^, respectively, while CO concentrations of 0.6–0.9, 0.8–0.9 and 0.5–0.6 mg/m^3^ are seen, respectively. The standard deviations of both NO_2_ and O_3_ display the largest in Hangzhou, the second in Qingdao, and the smallest in Xiamen. Generally, all six air pollutants in Qingdao and Hangzhou are higher than those in Xiamen, indicating heavier air pollution in Qingdao and Hangzhou. The NO_2_ concentrations are considerably more than SO_2_ in each of the cities, and this may suggest higher emissions of NO_2_ or non-linear oxidation reactions of SO_2_ and NO_2_ over eastern China [36]. The PM_2.5_ (PM_10_) concentrations from 2015 to 2018 are reduced by 32% (23%), 29% (19%), and 18% (11%) in Qingdao, Hangzhou and Xiamen, implying the effects of control policies on particulate matter by the governments. Compared to 2018, PM concentrations over Qingdao show increasing in 2019, probably indicating that a local control policy became loose. As precursor gases of secondary inorganic aerosols, the decrease of SO_2_ is favorable for PM_2.5_ reductions, while increasing NO_2_ contributes to increased PM_2.5_ from 2018 to 2019 in Qingdao. Meanwhile, the controls of SO_2_ are evident in Qingdao and Hangzhou, and 70% and 63% of SO_2_ was reduced from 2015 to 2019, respectively. Nonetheless, reductions of NO_2_, CO, and O_3_ are not obvious in the past five years, indicating that a control standard on gas pollution emissions need to be more integrated and rigorous in the eastern coast of China.

**Figure 3:**
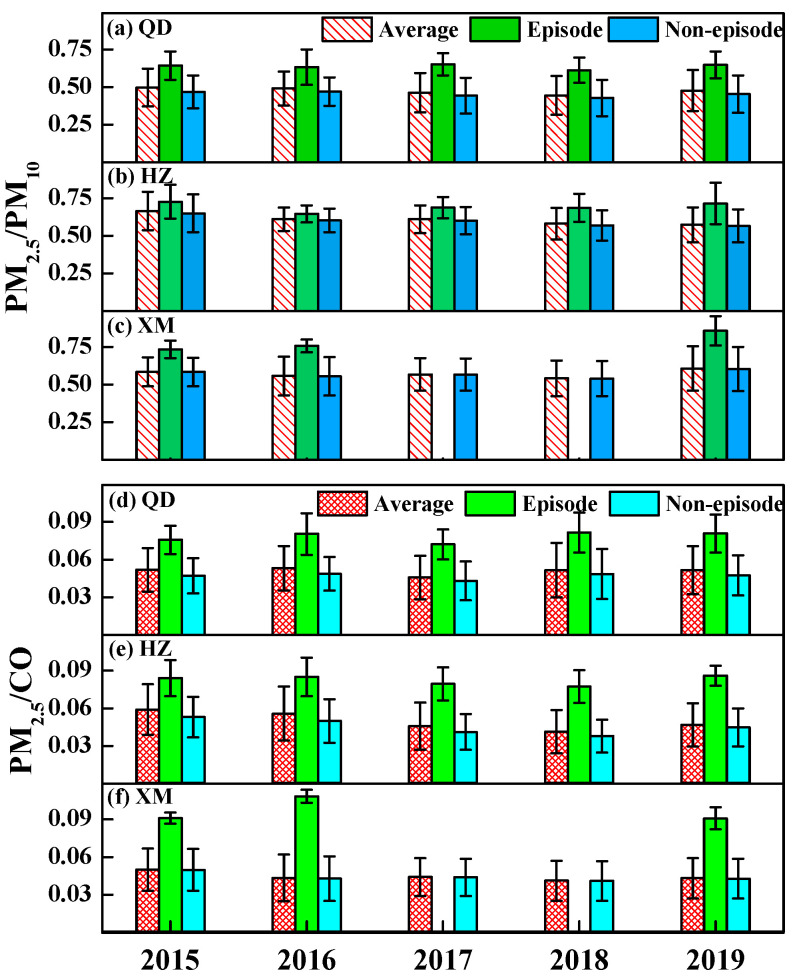
Inter-annual variations of PM_2.5_/PM_10_ (**a**–**c**) and PM_2.5_/CO (**d**–**f**) in Qingdao (QD), Hangzhou (HZ) and Xiamen (XM) for all (episode and non-episode) periods during 2015–2019. The error bar represents standard deviation.

The PM_2.5_/PM_10_ is a ratio indicator revealing particulate pollution characteristics and potential atmospheric processes, as fine and coarse particles have distinct source regions and physical-chemical properties [37]. Variations of annual average PM_2.5_/PM_10_ from 2015 to 2019 in these three metropolises of eastern coast China are illustrated in Figure 3. Yearly average PM_2.5_/PM_10_ ratios are seen in ranges of 0.44–0.50, 0.57–0.67, and 0.54–0.60 in Qingdao, Hangzhou and Xiamen, respectively during 2015 to 2019 (see Figure 3a–c). For a fixed year, Hangzhou has the highest mean PM_2.5_/PM_10_, followed by Xiamen, and Qingdao shows the lowest ratios, possibly indicating more primary PM sources in Qingdao compared to Hangzhou and Xiamen. The inter-annual variation trend of PM_2.5_/PM_10_ is positively related to that of PM_2.5_ or PM_10_ in Qingdao, and Hangzhou, showing a decrease from 2015 to 2018, and this indicates that reductions of PM in both cities are attributed to decreased emissions.

To quantify the secondary source contribution to PM_2.5_, PM_2.5_/CO is also considered because PM_2.5_ can be normalized by tracer CO and the impacts of meteorological condition and primary combustion source can be excluded [38]. The results of annual average PM_2.5_/CO from 2015 to 2019 over these three cities are shown in Figure 3d–f. It can be found that the yearly mean PM_2.5_/CO ratios during 2015 to 2019 are in ranges of 0.046–0.053, 0.042–0.059, and 0.041–0.050 over Qingdao, Hangzhou and Xiamen, respectively, indicating generally similar secondary aerosol contributions in metropolises of eastern coast China. It is interesting to notice that for Qingdao, the same PM_2.5_/CO values (i.e., 0.051) are observed in 2018 and 2019 whereas increased PM_2.5_/PM_10_ from 2018 to 2019 is seen. This suggests that the increase of PM_2.5_ from 2018 to 2019 in Qingdao is owing to primary particulate emissions, as levels of secondary aerosol sources are similar.

For haze pollution, aerosol number concentrations are high with numerous tiny particles floating in the atmosphere and affecting human health [39], and we consider pollution episode periods with PM_2.5_ > 75 μg/m^3^ [15]. Annual mean PM_2.5_/PM_10_ and PM_2.5_/CO during episodes and non-episodes from 2015 to 2019 are also shown in Figure 3. No pollution episodes occurred in Xiamen in 2017 and 2018. Obviously, higher PM_2.5_/PM_10_ ratios are observed during episodes, indicating more small particles associated with increased PM emissions. Meanwhile, PM_2.5_/CO ratios during episodes are higher than those in non-episode periods, and this implies more secondary aerosol contributions to PM_2.5_ in episode days. It is interesting to note that compared to 2017 and 2018, Xiamen suffers from PM episodes in 2019, whereas its PM loadings decrease in 2019. This suggests that Xiamen’s PM episodes in 2019 are not induced by increased emissions but possibly associated with stable weather conditions, which are conductive to particle accumulation and adverse to pollution diffusion.

**Figure 4:**
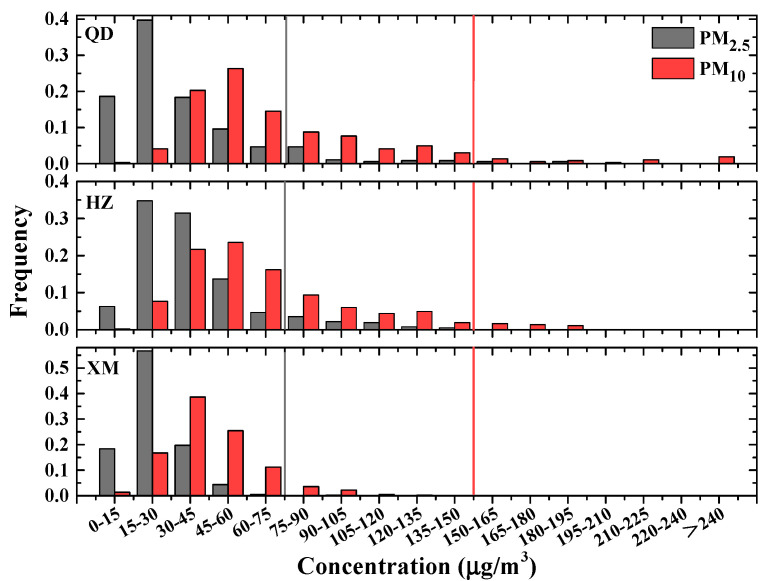
Frequency distributions of daily PM_2.5_ and PM_10_ in Qingdao (QD), Hangzhou (HZ) and Xiamen (XM) during 2018. The CAAQS Grade II of daily average for PM_2.5_ and PM_10_ are plotted as solid lines.

**Figure 5:**
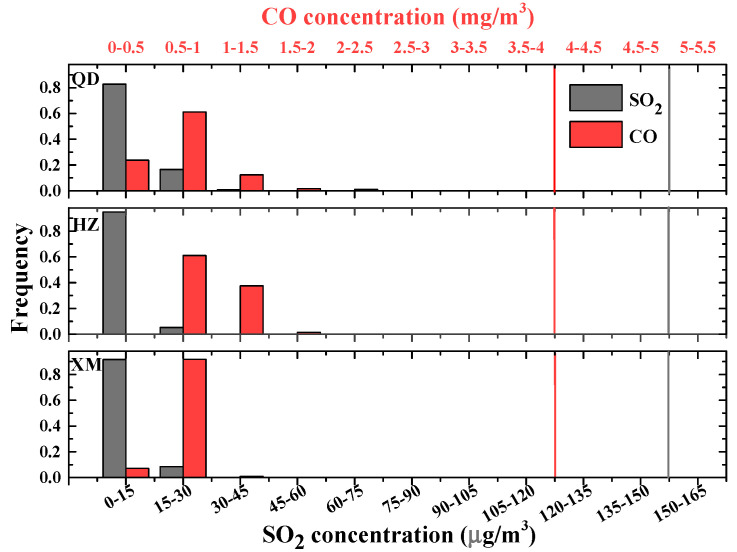
Frequency distributions of daily SO_2_ and CO in Qingdao (QD), Hangzhou (HZ) and Xiamen (XM) during 2018. The CAAQS Grade II of daily average for SO_2_ and CO are plotted as solid lines.

**Figure 6:**
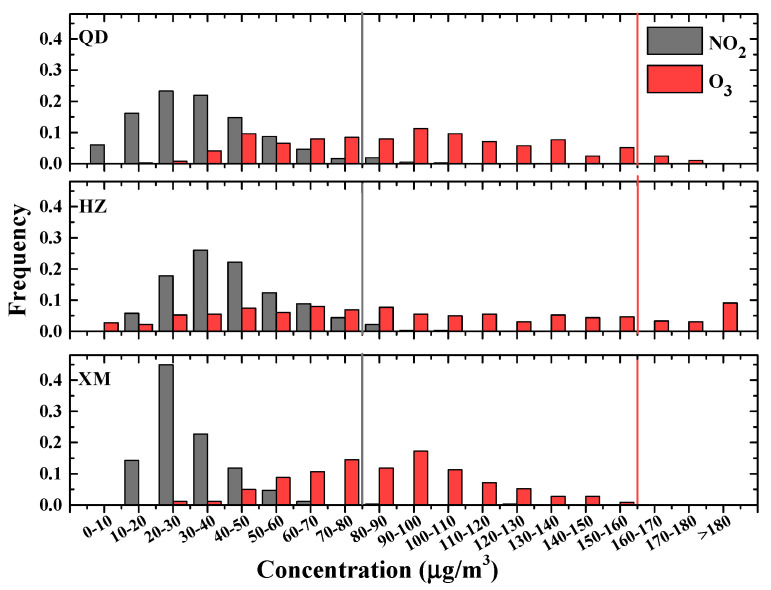
Frequency distributions of daily NO_2_ and O_3_ in Qingdao (QD), Hangzhou (HZ) and Xiamen (XM) during 2018. The CAAQS Grade II of daily average for NO_2_ and 8-h peak O_3_ are plotted as solid lines.

To further study the situations of air pollutants over three metropolises of eastern coast China, we investigate their frequency distributions during 2018, in which PM levels are low in recent five years. Figure 4 shows daily PM frequency distributions in three cities, while statistic results of gas pollutants are illustrated in Figure 5 and Figure 6. As shown in Figure 4, 9%, 5% and 0% of PM_2.5_ mass concentrations exceed the CAAQS-II daily standard (i.e., 75 μg/m^3^), whilst 6%, 4% and 0% of PM_10_ surpass Grade II of the concentration limits for 24 h mean (i.e., 150 μg/m^3^) in Qingdao, Hangzhou and Xiamen, respectively. This indicates that Qingdao and Hangzhou suffer from more PM pollutions whereas the situation in Xiamen is much better. Both SO_2_ and CO mass concentrations are below the CAAQS Grade II (150 μg/m^3^ and 4 mg/m^3^, respectively) in all three cities (see Figure 5). Mass concentrations of NO_2_ (O_3_) illustrated in Figure 6 show that 3% (5%), 3% (15%) and 0% (0%) surpass the CAAQS Grade II of 80 μg/m^3^ (160 μg/m^3^) in Qingdao, Hangzhou and Xiamen, respectively.

Overall, PM pollution situations over metropolises of eastern coast China have improved over the last five years with the concentrations decreasing about 20–30%, whereas caution may be taken in the reverse of PM levels in 2019 over Qingdao, indicating loose control of emissions policy by the local government. Meanwhile, gas pollutants (excepting SO_2_) generally show no evident reduction tendencies, and a more rigorous control standard on gaseous pollutants is needed because they are contributors to PM, e.g., NO_2_ being the precursor of secondary inorganic aerosols. Moreover, even for year 2018 with low air pollution levels, only SO_2_ and CO met the air quality standard, whereas <10% of PM_2.5_ and <15% of O_3_ fail to meet Grade II of the CAAQS over these metropolises of eastern coast China.

### 3.2. Seasonal and Diurnal Patterns of Air Pollutants

**Figure 7:**
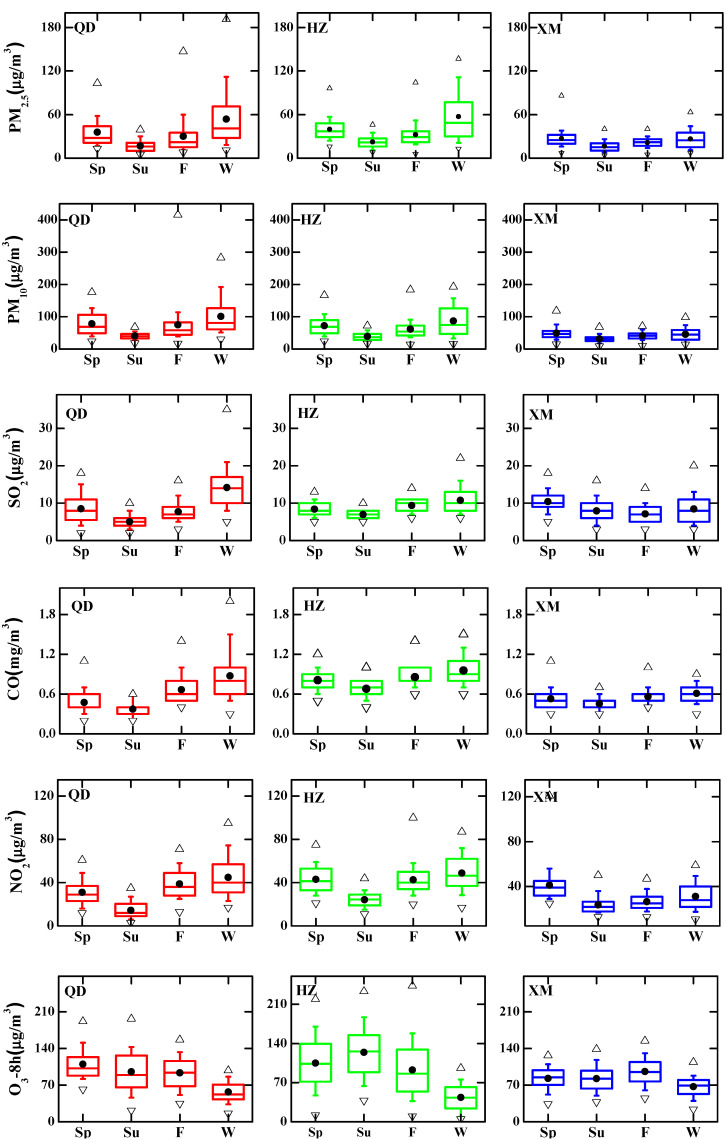
Seasonal variations of six pollutants in Qingdao (QD), Hangzhou (HZ) and Xiamen (XM) during 2018 (Sp, Su, F, and W indicate spring, summer, fall, and winter, respectively). The top and bottom whiskers show the 90th and 10th percentile while the upper and lower boundaries of the central box show 75th and 25th percentile. The middle line of the box represents median, the solid circle represents the arithmetic average, the positive triangle and inverted triangle represent max and min values, respectively.

For a better understanding of air pollution in eastern coastal China, PM and gaseous pollutants in terms of seasonal and diurnal variations are also investigated. Figure 7 illustrates regionally averaged seasonal variations of air pollutants over these metropolises of eastern coastal China. The mass concentrations of PM_2.5_ and PM_10_ exhibit similar seasonal cycles with low values in summer, owing to strong convection favorable for air pollutant dispersion and heavy precipitation scavenging particles [40]. The seasonal PM patterns in Qingdao and Hangzhou are similar, which are slightly different from that in Xiamen. The increased energy consumption associated with fossil fuels and biomass combustion could lead to a high level of PM mass concentrations in the cold season [32]. Severe PM loadings are seen in winter over Qingdao (53.7 μg/m^3^ for PM_2.5_ and 100.7 μg/m^3^ for PM_10_) and Hangzhou (57.1 μg/m^3^ for PM_2.5_ and 86.4 μg/m^3^ for PM_10_), in association with combined effects of strong particle emissions and stable weather conditions in winter [41,42], whereas Xiamen has a maritime climate with mild winter temperatures, showing high PM levels (26.4 μg/m^3^ for PM_2.5_ and 45.5 μg/m^3^ for PM_10_) in spring. For all four seasons, Qingdao shows the highest PM_10_ concentrations and Hangzhou exhibits largest PM_2.5_ levels among the three cities, indicating the impacts of emissions on PM loadings in Qingdao and Hangzhou. The wintertime PM mass concentrations in Qingdao and Hangzhou are a factor of ~2 larger than that in Xiamen, indicating lower health risk due to particulate exposure in winter of Xiamen.

Local NO_2_ emissions generally originate from vehicles, power stations, and industries with the resulting energy exhaust, among which vehicles account for the most [43]. As shown in Figure 7, NO_2_ concentrations increase remarkably from summer to winter over Qingdao (14.5–44.9 μg/m^3^) and Hangzhou (24.3–48.9 μg/m^3^) or from summer to next spring in Xiamen (23.8–41.2 μg/m^3^). SO_2_ discharge is primarily from fossil fuel combustion [4]. Similar with the distinguishable seasonal pattern of NO_2_, SO_2_ has the largest seasonal variation in Qingdao, with the concentration in winter (14.2 μg/m^3^) nearly three times higher than in summer (5.0 μg/m^3^), while the least in Xiamen, with the difference of ~3.3 μg/m^3^ between the highest spring (10.4 μg/m^3^) and lowest autumn (7.1 μg/m^3^) concentrations. Mean values of CO concentrations in each season in all three cities are quite low, no more than 1 mg/m^3^. CO concentrations were comparable in spring, summer, and fall but winter has a slightly higher value and wider distribution. As a whole, high NO_2_, SO_2_ and CO concentrations are consistent with high PM values during the same period. Stagnant weather systems occur more frequently in the low-temperature season; these systems trap pollutants near the surface, and thus result in high concentration levels as a result of the accumulation process.

Under the conditions of photochemical reactions, primary precursors, such as volatile organic compounds (VOC), NO_x_ and CO, form secondary pollutant O_3_. O_3_ concentration is related to sunshine time, solar radiation intensity and temperature. O_3_ exhibits an opposite seasonal trend from other gaseous and PM pollutants, and winter is the best season with lowest O_3_ concentrations ranging from 43.3 to 67.2 μg/m^3^. The worst seasons for Qingdao, Hangzhou, and Xiamen are the spring (109.8 μg/m^3^), summer (124.1 μg/m^3^) and autumn (96.0 μg/m^3^), respectively. Pollution-laden airflow brought by the prevailing wind, coupled with the stable and warm weather, contributes to high O_3_ amplitudes in inconsistent seasons.

**Figure 8:**
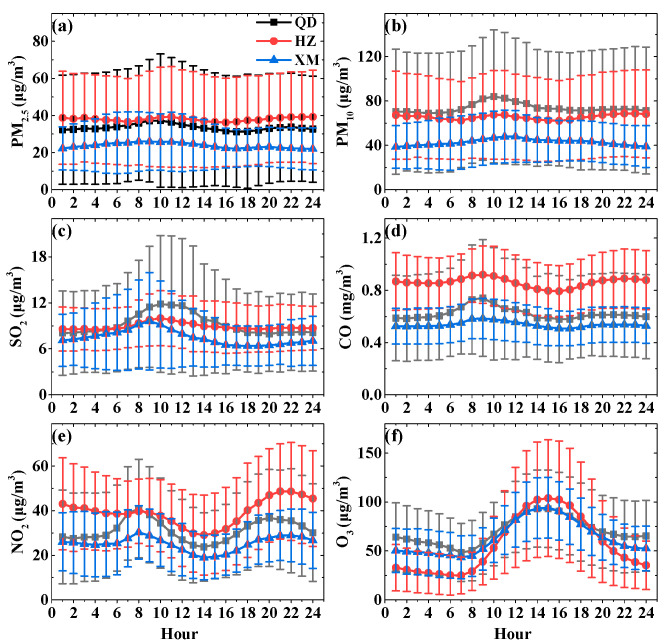
Diurnal variations and corresponding standard deviation of PM_2.5_ (**a**), PM_10_ (**b**), SO_2_ (**c**), CO (**d**), NO_2_ (**e**), and O_3_-1h (**f**) in Qingdao (QD), Hangzhou (HZ), and Xiamen (XM) during 2018.

Figure 8 illustrates diurnal variations of six air pollutants in Qingdao, Hangzhou, and Xiamen. To allow more accurate and objective conclusions about the main sources of pollutant emission (transportation, industry, and heating sources) in the analyzed urban areas, the analysis of diurnal variations are also carried out for the seasons separately in Figure S1.

Both PM and gas pollutants (except O_3_) show dramatically higher concentrations and stronger diurnal variations in Qingdao and Hangzhou than that in Xiamen. Obviously, the seasonal curves show a dispersive distribution. These pollutants start to increase slowly around 6:00–7:00 and reach their highest concentration in the morning rush hours of 9:00–10:00. The flat valley appeared in the early afternoon (14:00–15:00), primarily due to the relatively high height of the planetary boundary layer, which is conducive to the diffusion of air pollutants. Concentrations increase again after 18:00 pm and remain high until midnight. Pollutant emissions from increased cross-city truck traffic, which is controlled during daytime, may explain the high pollutant accumulation during the evening rush hours. As shown in Figure S1, for the morning peak, the winter is later than spring, summer, and fall whereas for the evening peak, the former is earlier. The peaks appear at night during wintertime, especially for PM in Qingdao and Hangzhou, are more pronounced than other seasons. It may be explained by starting to use coal and other fuels for heating and relative stability weather, causing air pollution more seriously in the cold season. Compared with Qingdao and Hangzhou, the differences of six pollutants in Xiamen in the four seasons are weaker.

Interestingly, O_3_ distribution demonstrates only slight differences in terms of both diurnal variability and concentrations among the three coastal cities. The O_3_ exhibits a unimodal distribution. Its concentrations begin to increase at 8:00 and appear as an obvious peak at ~15:00 in the afternoon. With the sun setting, the photochemical process weakens and the O_3_ concentrations decrease as well, especially at night in winter.

From the above analysis, the seasonal and diurnal patterns of air pollutants are of great significance to understand the impact of meteorological parameters and potential emission sources.

### 3.3. Pollutants Source Analysis Based on Backward Trajectory and CWT

**Figure 9:**
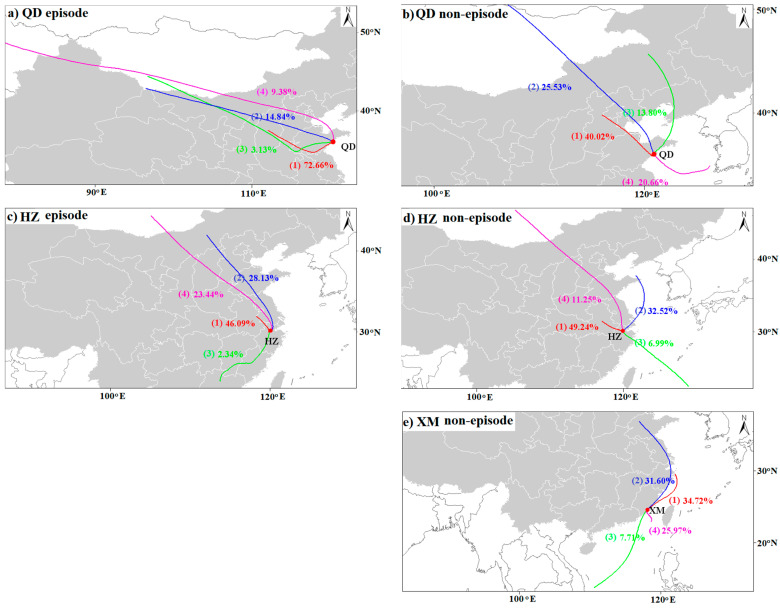
72-h air-mass backward trajectories for episode (**a**,**c**) and non-episode (**b**,**d**,**e**) periods in Qingdao (**a**,**b**), Hangzhou (**c**,**d**), and Xiamen (**e**) during 2018. No pollution episodes occurred in Xiamen in 2018.

**Figure 10:**
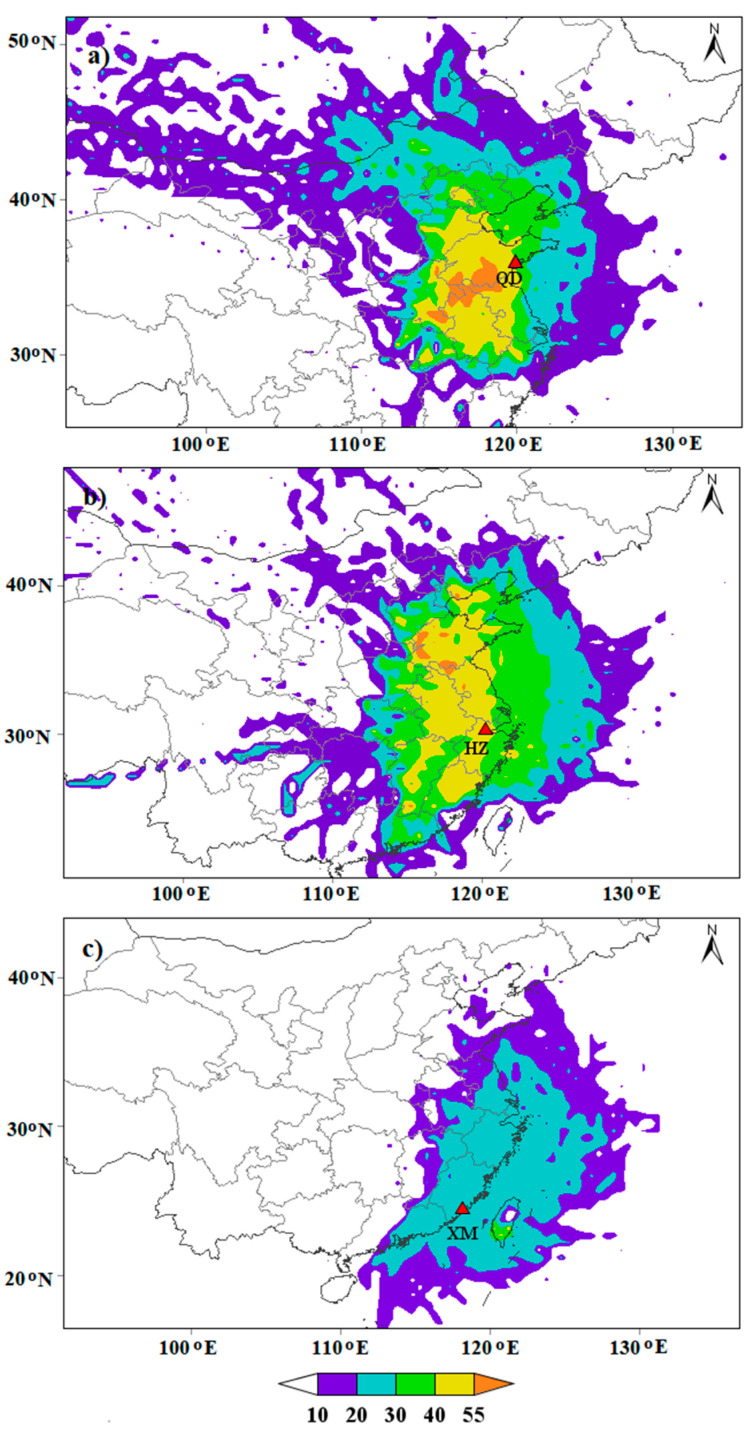
Concentration-weighted trajectory (CWT) maps for PM_2.5_ in Qingdao (**a**), Hangzhou (**b**), and Xiamen (**c**) during 2018. The red solid triangle denotes the center of the studied city, and the CWT values are displayed in color.

To evaluate the impact of local emission and regional transportation of pollutants on air quality, the 72-h air-mass backward trajectories of Qingdao, Hangzhou and Xiamen are calculated by the TrajStat model. The trajectories for the three cities are clustered during episode and non-episode periods in 2018, respectively (Figure 9). The CWT model is applied to weight trajectories with related PM_2.5_ concentrations to identify the relative contribution of potential source areas (Figure 10).

As displayed in Figure 9, the transport pathways and distance of the trajectories depict obvious differences in episode and non-episode days. Qingdao is located in the east of Shandong Peninsula. Three major directions of trajectories are identified during episode periods: southwest (Cluster 1 and 3), northwest (Cluster 2), and north (Cluster 4). Cluster 1, accounting for 73% of all air masses, originates in Shanxi Province and travels across southern Hebei and west Shandong Provinces before penetrating into Qingdao. Clusters 2 and 3 account for 15% and 3% of air masses, begin in the Mongolia and Inner Mongolia, respectively. Especially in spring and winter, the prevailing westerly wind (southwest and northwest) always carries the aerosol particles from highly polluted regions to Qingdao to make the pollution in this area worse. Additionally, its air quality also is affected by the long-distance transmission of air masses from Xinjiang (9%). During the non-episode periods, except that Cluster 1 (40%) is an inland trajectory cluster, the other three clusters stem from Yellow Sea (Cluster 4) or pass through the Bohai Sea (Cluster 2 and 3). The input of relatively clean marine air masses is beneficial for the air pollution dispersion, ultimately resulting in PM_2.5_ concentrations below the 75 μg/m^3^ limit. The analysis of air quality in urban agglomeration areas is mainly related to local emission sources and nearby cities. Pollutants emitted in other, particularly remote areas are largely dispersed in the air. The CWT model identifies that the local source as well as its adjacent area including the west of Shandong, north of Jiangsu, and Anhui provinces are major potential sources areas of ambient PM_2.5_, revealing that cross-boundary transport plays a vital role in PM pollution. Qingdao also has a weak contribution to PM_2.5_ from distant Inner Mongolia and Shanxi with CWT, between 20 and 40 μg/m^3^. The Bohai Sea, the Yellow Sea, and nearby coastal region are also potential sources of PM_2.5_ with CWT below 30 μg/m^3^.

Hangzhou is located on the south of the Yangtze River Delta and is also mainly affected by the air mass trajectories in three directions during the episode period: northwest (Cluster 1), north (Cluster 2 and 4), and southwest (Cluster 3). Cluster 1, with a ratio of 46%, represents influences from the adjacent region of the southern Anhui and Jiangsu provinces, which belongs to short-distance air transport or local emissions. Clusters 2 and 4 are derived from eastern and central Inner Mongolia, and then move through northern Shanxi, southern Hebei, west Shandong and Jiangsu Provinces prior to arriving at the destination, accounting for 28% and 23% of total trajectories, respectively. The proportion of the last type of cluster accounts for only 2%, and originates from southern Jiangxi. Similar to the trajectory of Qingdao, two clusters from the marine sources with cleaner air masses are observed for Hangzhou during a non-episode period. One type of trajectory (33%) comes from the Yellow Sea and enters Hangzhou city through the Hangzhou Bay; the other type (7%) starts from the remote South China Sea and travels a long distance over the ocean. High CWT to PM_2.5_ concentrations for Hangzhou are observed in the local city, the north of Zhejiang, most parts of Anhui and Jiangsu, and west of Shangdong province.

Xiamen is the coastal city of the Pearl River Delta with no episode period. During the non-episode days, almost 68% of the airmasses come from the ocean, and its ratio is much larger than that for Qingdao (35%) and Hangzhou (40%). In addition, 32% of airmasses are transported from the north to the south part of China, directly sweeping over the Yangtze River Delta including the cities of Jiangsu, Zhejiang, and the megacity of Shanghai. Compared with Qingdao and Hangzhou, CWT values are significantly lower in Xiamen with the value below 30 μg/m^3^.

In brief, the back-trajectory and CWT results demonstrate that the pollution sources of Qingdao and Hangzhou are mainly land emissions, followed by the marine influence. Xiamen enjoyed non-episode status throughout the year 2018. As PM pollution breaks out, the polluted airmasses basically come from inland, especially pollution emission from local and adjacent cities and provinces. Improving air quality is not a merely local issue, but a regional issue.

## 4. Conclusions

We used hourly monitoring data in three metropolises, i.e., Qingdao, Hangzhou, and Xiamen from 2015 to 2019, to evaluate the current situations of PM and trace gases for the eastern coast of China. In general, PM pollution situations improved in recent years, while caution may be taken in the reverse of PM levels in 2019 over Qingdao because of loosening of the control policy of emissions by the local government. As for gas pollutants, no obvious reduction tendencies are observed except SO_2_, and stricter standards need to be implemented. Even for year 2018 with low air pollution levels, only SO_2_ and CO meet the Grade II of China Air Quality Daily Standards, whereas <10% of PM_2.5_ and <15% of O_3_ surpass Grade II standards over these metropolises.

The annual average concentrations of PM_2.5_, CO and NO_2_ follow the order of Hangzhou > Qingdao > Xiamen, while the annual concentration of PM_10_ and SO_2_ follow the order of Qingdao > Hangzhou > Xiamen. The data of PM_2.5_/PM_10_ and PM_2.5_/CO state clearly that the static meteorological conditions as well as the formation of secondary aerosols have important impacts on air pollutant aggravation when haze episodes occur. PM, CO, SO_2_, and NO_2_ have distinct seasonal trends with the maximum in winter and the minimum in the summer whereas O_3_ shows the completely opposite pattern for Hangzhou and Qingdao. Xiamen belongs to the subtropical marine monsoon climate with mild winter temperature, exhibiting high levels of PM and trace gases in spring. The diurnal variation curves show obvious peaks and troughs. It reaches the peaks at 9:00–10:00 and 18:00 to midnight for the pollutants with the exception of O_3_, whereas a completely adverse trend pattern is observed for O_3_ with a peak ~15:00.

Improving people’s understanding of transport routes and potential source regions of air pollution is essential for formulating and implementing pollution control policies. The backward trajectories and CWT models are used to explore the transport pathways and source distribution. Trajectories from local and nearby region associated local emissions contribute the most to the level of PM_2.5_ concentrations, and show correspondingly higher CWT values. Besides of local emission cuts, implementing a unified air pollution prevention policy and strengthening regional cooperation can effectively alleviate air pollution in coastal cities.

## Data Availability

Data openly available in a public repository. The data download link is provided in the ‘*Data and Methodology*’ section (References [25,26]).

## Supplementary Materials

The following are available online at https://www.mdpi.com/article/10.3390/ijerph192215332/s1.
