# Supplementary material for: Air Pollutants in Metropolises of Eastern Coastal China
Source: Int J Environ Res Public Health. 2022 Nov 20;19(22):15332. doi: 10.3390/ijerph192215332 (PMC9691249; doi:10.3390/ijerph192215332)
Supplement: Supplementary file 1 [file ijerph-19-15332-s001.zip › ijerph-2013552-supplementary.pdf]

Air Pollutants in Metropolises of Eastern Coastal China

Mao Mao[1,2], Liuxintian Rao[1], Huan Jiang[2], Siqi He[1], Xiaolin Zhang[1,2,*]

[1] School of Atmosphere and Remote Sensing, Wuxi University, Wuxi 214105, China

[2] Key Laboratory for Aerosol-Cloud-Precipitation of China Meteorological Administration, School of Atmospheric Physics, Nanjing University of Information Science & Technology, Nanjing 210044, China

* Corresponding: xlnzhang@nuist.edu.cn

Table S1. Detailed information of National Environmental Monitoring stations in Qingdao, Hangzhou and Xiamen.

**Station**	**Description**	Longitude	Latitude
Qingdao (9)			
YK	Yangkou	120.66	36.24
LC	Licang	120.39	36.18
SB	Sibei	120.34	36.06
SN_E	Eastern Shinan	120.41	36.06
SF	Sifang	120.36	36.10
SN_W	Western Shinan	120.29	36.05
LS	Laoshan	120.45	36.08
HD	Huangdao	120.19	36.30
CY	Chengyang	120.40	36.24
Hangzhou (11)			
BJ	Binjiang	120.21	30.21
XX	Xixi	120.06	30.27
QDH	Qiandaohu	119.02	29.63
XS	Xiasha	120.34	30.30
WLQ	Wolongqiao	120.12	30.24
ZJND	Zhejiangnongda	120.19	30.26
ZHWQ	Zhaohuiwuqu	120.15	30.28
HMXX	hemuxiaoxue	120.12	30.31
LPZ	Linpingzhen	120.30	30.41
CXZ	Chengxiangzhen	120.27	30.18
YQ	Yunqi	120.08	30.18
Xiamen (3)			

XD	Xidong	118.15	24.81
HW	Hongwen	118.15	24.47
GLY	Gulangyu	118.06	24.44

Table S2. Annual mean concentrations of six criteria air pollutants with standard deviations in Qingdao, Hangzhou and Xiamen during 2015–2019 (units are μg/m³ for $PM_{2.5}$, PM_{10}, NO_2, SO_2 and O_3, and mg/m³ for CO)[a].

	City	2015	2016	2017	2018	2019	CAAQS II[b]
$PM_{2.5}$	Qingdao	51.0±38.1	44.7±33.7	37.8±29.2	34.7±28.6	39.5±34.2	75
	Hangzhou	55.4±31.0	48.9±28.7	44.6±26.5	39.2±23.9	38.0±21.5	
	Xiamen	29.6±13.2	28.0±16.1	27.0±13.8	24.1±11.3	24.1±11.6	
PM_{10}	Qingdao	97.5±53.4	87.2±54.4	77.0±42.6	74.7±49.4	78.9±51.9	150
	Hangzhou	83.3±42.1	79.7±44.8	71.9±38.1	67.1±36.0	66.4±33.9	
	Xiamen	50.3±18.7	49.2±23.5	48.0±23.2	44.6±18.6	39.9±16.7	
SO_2	Qingdao	27.4±15.5	21.6±12.8	15.2±8.7	9.4±5.4	8.1±4.6	150
	Hangzhou	15.6±6.9	11.8±4.9	11.1±3.9	9.4±2.8	6.7±2.0	
	Xiamen	9.3±4.0	10.8±4.9	11.0±4.8	9.1±3.6	5.6±2.0	
CO	Qingdao	0.91±0.40	0.78±0.37	0.77±0.35	0.64±0.33	0.70±0.37	4
	Hangzhou	0.90±0.27	0.84±0.24	0.93±0.23	0.91±0.20	0.78±0.21	
	Xiamen	0.60±0.16	0.63±0.18	0.59±0.15	0.58±0.14	0.56±0.15	
NO_2	Qingdao	33.4±15.2	33.4±14.9	37.8±16.9	33.8±18.2	35.6±19.2	80
	Hangzhou	46.3±16.6	44.8±18.5	45.4±17.1	41.9±16.6	41.2±15.2	
	Xiamen	28.5±9.9	30.9±13.1	32.1±15.8	29.9±11.8	23.2±10.9	
O_3-8h	Qingdao	97.7±39.6	95.0±35.5	104.4±47.1	94.8±38.8	96.2±41.3	160
	Hangzhou	94.4±50.2	95.9±50.2	100.2±57.2	97.3±55.9	99.1±55.6	
	Xiamen	66.4±21.3	65.6±26.3	79.3±28.9	86.9±26.3	92.1±30.9	

[a] The error denotes a standard deviation. [b] The grade II of Chinese Ambient Air Quality Standards for daily mean.

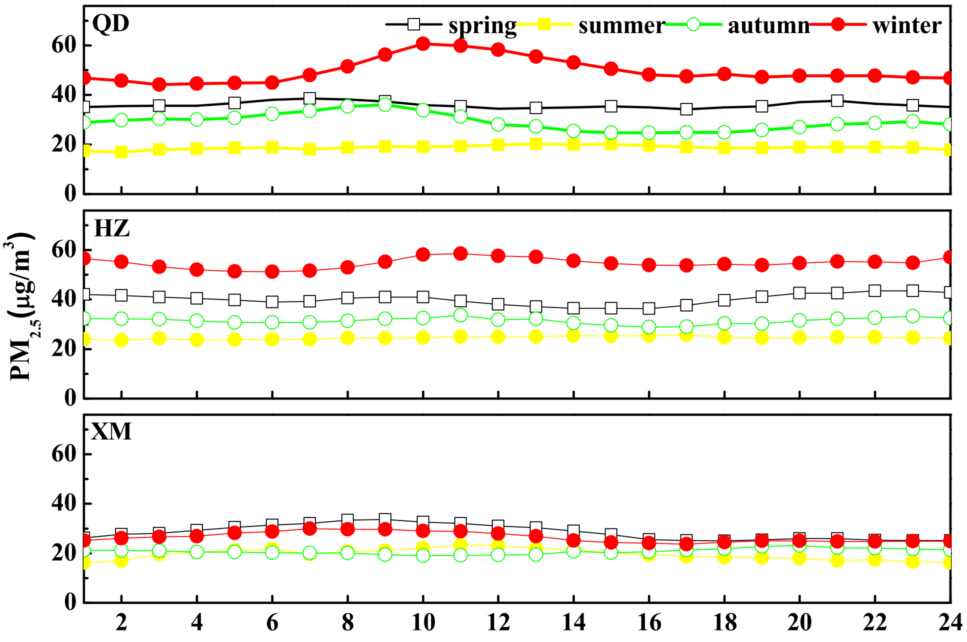

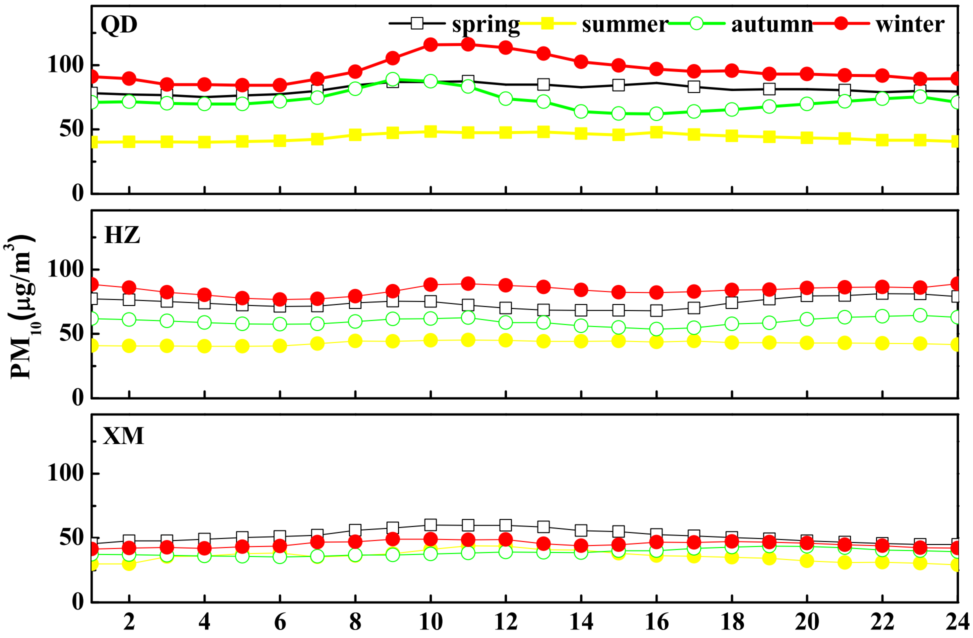
QD
spring
summer
autumn
winter
HZ
XM
PM$_{10}$(μg/m^3)
0
50
100
2
4
6
8
10
12
14
16
18
20
22
24

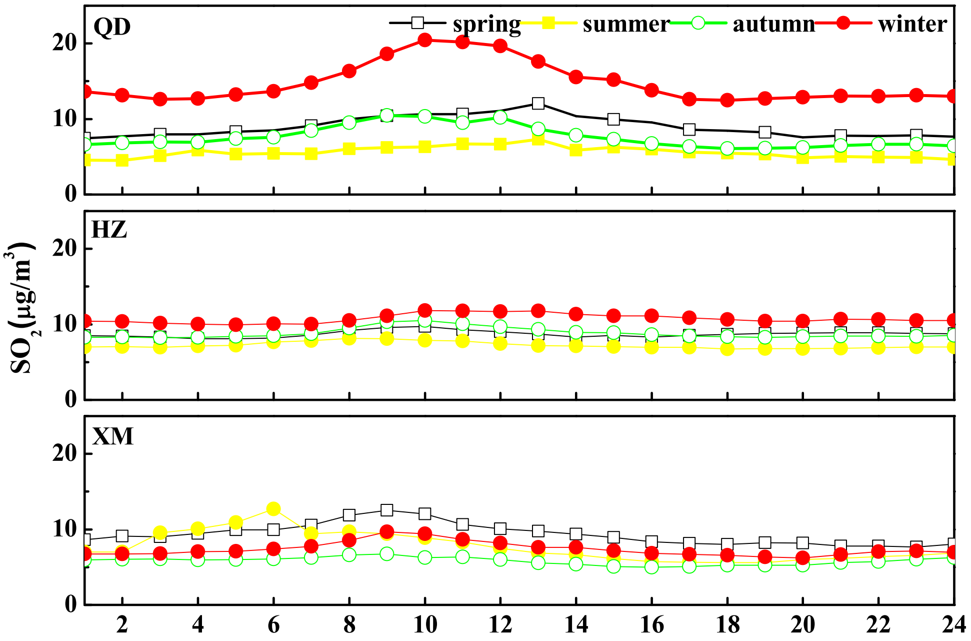
QD
spring
summer
autumn
winter
HZ
XM
SO$_2$(μg/m^3)
0
10
20
2
4
6
8
10
12
14
16
18
20
22
24

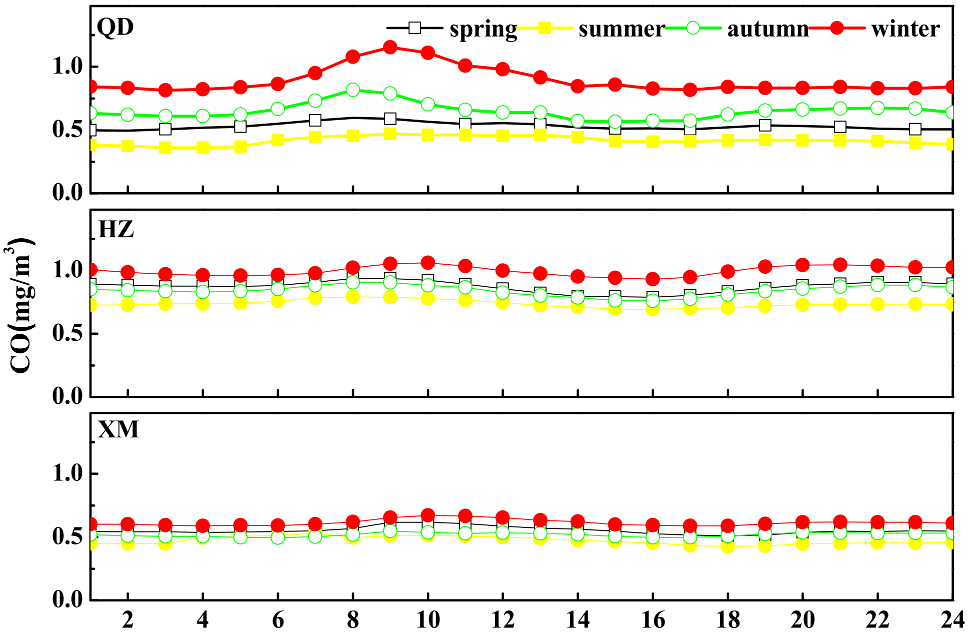
QD
spring
summer
autumn
winter
HZ
XM
CO(mg/m3)
0.0
0.5
1.0
2
4
6
8
10
12
14
16
18
20
22
24

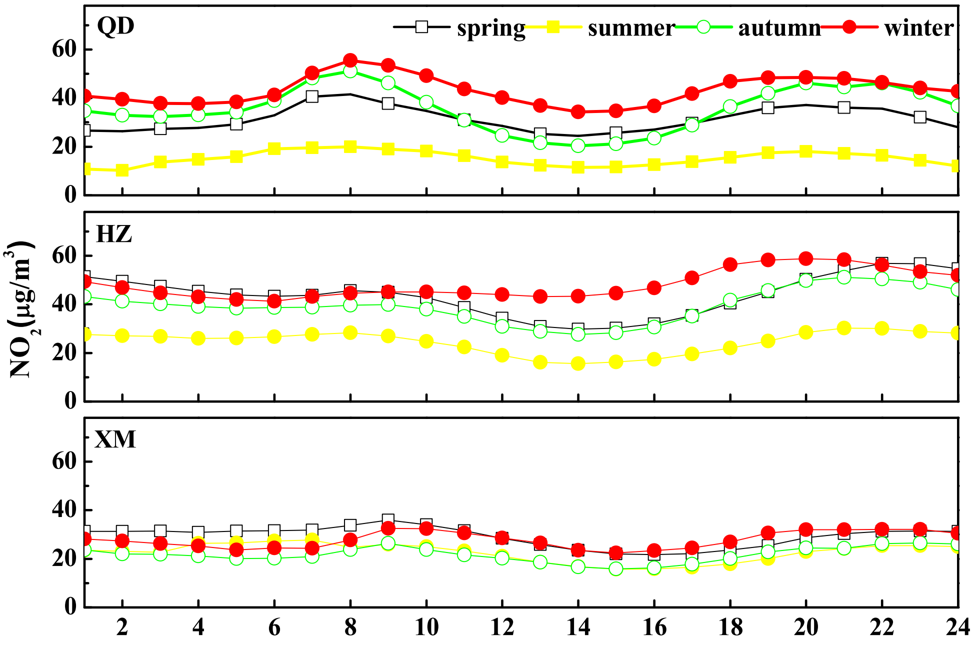
QD
spring
summer
autumn
winter
HZ
XM
NO2(μg/m3)
0
20
40
60
2
4
6
8
10
12
14
16
18
20
22
24

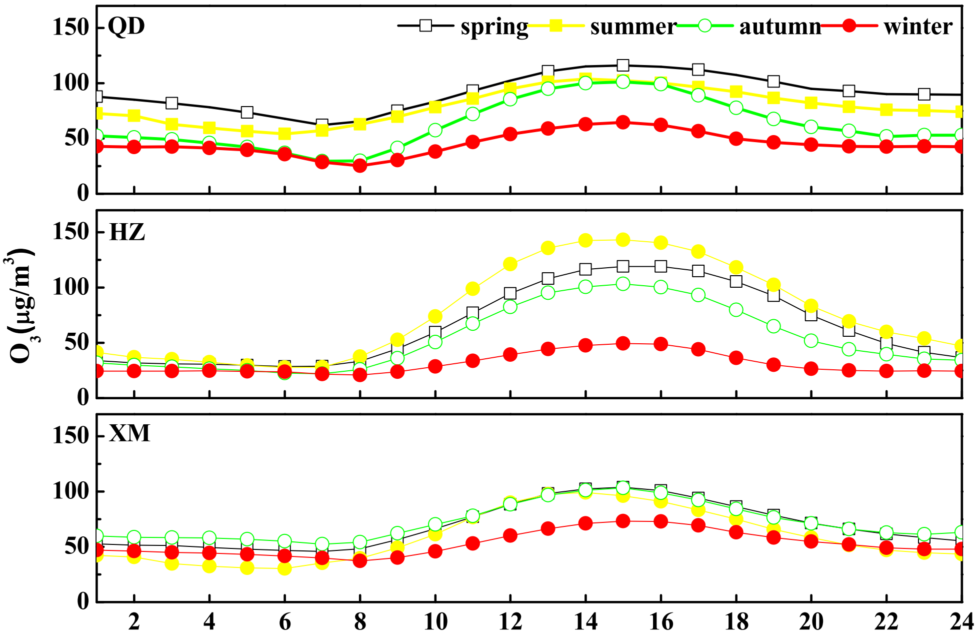

Figure S1. Diurnal variations of hourly $PM_{2.5}$, PM_{10}, SO_2, CO, NO_2, and O_3-1h mass concentrations in pollutants in Qingdao (QD), Hangzhou (HZ) and Xiamen (XM) during four seasons. Note that here is the hourly variation of O_3, not the 8 h peak O_3 (O_3-8h).
